# Supplementary material for: Physical activity questionnaire for older children (PAQ-C): Arabic translation, cross-cultural adaptation, and psychometric validation in school-aged children in Saudi Arabia
Source: PeerJ. 2022 Apr 12;10:e13237. doi: 10.7717/peerj.13237 (PMC9012174; doi:10.7717/peerj.13237)
Supplement: Supplemental Information 2 [file peerj-10-13237-s002.pdf]

Raw Data – Code Book

1. S.No – Serial number allotted to each participant
2. Age – in years
3. Age group – (1= 8 to 10 years), (2 = 10 to 12 years), (3 = 12 to 14 years)
4. Gender – (1= Boy, 2 = Girl)
5. Grade – Actual Grade of the Participant
6. Height – in Centimetres (cm)
7. Weight – in Kilograms (kg)
8. BMI – Body mass index in kg/m^2
9. Han-Dom- Hand dominance (1 = Left and 2 = Right)
10. Physical activity questionnaire items (PAQ-C)
    1) P1_7 days- Physical activity in your spare time: Have you done any of the following activities in the past 7 days (last week)?
    2) P2_PE - In the last 7 days, during your physical education (PE) classes, how often were you very active (playing hard, running, jumping, throwing)?
    3) P3_recess - In the last 7 days, what did you do most of the time *at recess*?
    4) P4_Lunch - In the last 7 days, what did you normally do *at lunch* (besides eating lunch)?
    5) P5_Aft Sch- In the last 7 days, on how many days *right after school*, did you do sports, dance, or play games in which you were very active?
    6) P6_Eveng - In the last 7 days, on how many *evenings* did you do sports, dance, or play games in which you were very active?
    7) P7_Wk end- *On the last weekend*, how many times did you do sports, dance, or play games in which you were very active?
    8) P8_Best- Which *one* of the following describes you best for the last 7 days? Read *all five* statements before deciding on the *one* answer that describes you.
    9) P9_Score - Mark how often you did physical activity (like playing sports, games, doing dance, or any other physical activity) for each day last week.
    10) P10_Sick - Were you sick last week, or did anything prevent you from doing your normal physical activities? – (Yes -1, No -0)
    11) PTot Scor – Total score for P1 to P9
    12) PSumm Score – PAQ – C summary score

Scoring Instructions

Overall process - Find an activity score between 1 and 5 for each item (excluding item 10)

Five Easy Steps

1) *Item 1 (Spare time activity)*

- Take the mean of all activities ("no" activity being a 1, "7 times or more" being a 5) on the activity checklist to form a composite score for item 1.

2) *Items 2 to 8 (PE, recess, lunch, right after school, evening, weekends, and describes you best)*

- The answers for each item start from the lowest activity response and progress to the highest activity response

- Simply use the reported value that is checked off for each item (the lowest activity response being a 1 and the highest activity response being a 5).

3) *Item 9*

- Take the mean of all days of the week ("none" being a 1, "very often" being a 5) to form a composite score for item 9.

4) *Item 10*

- Can be used to identify students who had unusual activity during the previous week, but this question is **NOT** used as part of the summary activity score.

5) *How to calculate the final PAQ-C activity summary score*

- Once you have a value from 1 to 5 for each of the 9 items (items 1 to 9) used in the physical activity composite score, you simply take the mean of these 9 items, which results in the final PAQ-C activity summary score.

- A score of 1 indicates low physical activity, whereas a score of 5 indicates high physical activity.

The English version of the PAQ-C is attached below for further reference

Physical Activity Questionnaire (Elementary School)

Name:_________________________ Age:___________

Sex: M_______ F_______ Grade:__________

Teacher:_______________________

We are trying to find out about your level of physical activity from ***the last 7 days*** (in the last week). This includes sports or dance that make you sweat or make your legs feel tired, or games that make you breathe hard, like tag, skipping, running, climbing, and others.

Remember:

1. There are no right and wrong answers — this is not a test.
2. Please answer all the questions as honestly and accurately as you can — this is very important.

---

1. Physical activity in your spare time: Have you done any of the following activities in the past 7 days (last week)? If yes, how many times? (Mark only one circle per row.)

	No	1-2	3-4	5-6	7 times or more
Skipping	❍	❍	❍	❍	❍
Rowing/canoeing	❍	❍	❍	❍	❍
In-line skating	❍	❍	❍	❍	❍
Tag	❍	❍	❍	❍	❍
Walking for exercise	❍	❍	❍	❍	❍
Bicycling	❍	❍	❍	❍	❍
Jogging or running	❍	❍	❍	❍	❍
Aerobics	❍	❍	❍	❍	❍
Swimming	❍	❍	❍	❍	❍
Baseball, softball	❍	❍	❍	❍	❍
Dance	❍	❍	❍	❍	❍
Football	❍	❍	❍	❍	❍
Badminton	❍	❍	❍	❍	❍
Skateboarding	❍	❍	❍	❍	❍
Soccer	❍	❍	❍	❍	❍
Street hockey	❍	❍	❍	❍	❍
Volleyball	❍	❍	❍	❍	❍
Floor hockey	❍	❍	❍	❍	❍
Basketball	❍	❍	❍	❍	❍
Ice skating	❍	❍	❍	❍	❍
Cross-country skiing	❍	❍	❍	❍	❍
Ice hockey/ringette	❍	❍	❍	❍	❍
Other:					
___________________________	❍	❍	❍	❍	❍
___________________________	❍	❍	❍	❍	❍

2. In the last 7 days, during your physical education (PE) classes, how often were you very active (playing hard, running, jumping, throwing)? (Check one only.)

I don't do PE ....❍
Hardly ever ....❍
Sometimes ....❍
Quite often ....❍
Always ....❍

3. In the last 7 days, what did you do most of the time *at recess*? (Check one only.)

Sat down (talking, reading, doing schoolwork)……❍
Stood around or walked around ....❍
Ran or played a little bit ....❍
Ran around and played quite a bit ....❍
Ran and played hard most of the time ....❍

4. In the last 7 days, what did you normally do *at lunch* (besides eating lunch)? (Check one only.)

Sat down (talking, reading, doing schoolwork)…....❍
Stood around or walked around ....❍
Ran or played a little bit ....❍
Ran around and played quite a bit ....❍
Ran and played hard most of the time ....❍

5. In the last 7 days, on how many days *right after school*, did you do sports, dance, or play games in which you were very active? (Check one only.)

None ....❍
1 time last week ....❍
2 or 3 times last week ....❍
4 times last week ....❍
5 times last week ....❍

6. In the last 7 days, on how many *evenings* did you do sports, dance, or play games in which you were very active? (Check one only.)

None ....❍
1 time last week ....❍
2 or 3 times last week ....❍
4 or 5 last week ....❍
6 or 7 times last week ....❍

7. *On the last weekend*, how many times did you do sports, dance, or play games in which you were very active? (Check one only.)

None .... ❍
1 time .... ❍
2 — 3 times .... ❍
4 — 5 times .... ❍
6 or more times .... ❍

8. Which *one* of the following describes you best for the last 7 days? Read *all five* statements before deciding on the *one* answer that describes you.

A. All or most of my free time was spent doing things that involve little physical effort .... ❍

B. I sometimes (1 — 2 times last week) did physical things in my free time (e.g. played sports, went running, swimming, bike riding, did aerobics) .... ❍

C. I often (3 — 4 times last week) did physical things in my free time .... ❍

D. I quite often (5 — 6 times last week) did physical things in my free time .... ❍

E. I very often (7 or more times last week) did physical things in my free time .... ❍

9. Mark how often you did physical activity (like playing sports, games, doing dance, or any other physical activity) for each day last week.

	None	Little bit	Medium	Often	Very often
Monday	❍	❍	❍	❍	❍
Tuesday	❍	❍	❍	❍	❍
Wednesday	❍	❍	❍	❍	❍
Thursday	❍	❍	❍	❍	❍
Friday	❍	❍	❍	❍	❍
Saturday	❍	❍	❍	❍	❍
Sunday	❍	❍	❍	❍	❍

10. Were you sick last week, or did anything prevent you from doing your normal physical activities? (Check one.)

Yes .... ❍
No .... ❍

If Yes, what prevented you? ____________________________________
