# Supplementary material for: Physical activity questionnaire for older children (PAQ-C): Arabic translation, cross-cultural adaptation, and psychometric validation in school-aged children in Saudi Arabia
Source: PeerJ. 2022 Apr 12;10:e13237. doi: 10.7717/peerj.13237 (PMC9012174; doi:10.7717/peerj.13237)
Supplement: Supplemental Information 3 [file peerj-10-13237-s003.pdf]

Supplementary File – Arabic Version of Physical Activity Questionnaire for Older Children (PAQ-C)

استبيان النشاط البدني للأطفال الأكبر سنا (PAQ-C)

الأسم : .... السن : ....

النوع : ذكر .... أنثى .... المستوى : ....

المدرس : ....

نحن نحاول معرفة مستوى نشاطك البدني من آخر 7 أيام (في الأسبوع الأخير). وهذا يشمل الرياضة أو الرقص الذي يجعلك تعرق أو تجعل ساقيك يشعران بالتعب ، أو الألعاب التي تجعلك تتنفس بصعوبة ، مثل القفز والجري والتسلق وغيرها.

تــذكـــر

1. **لا توجد إجابات صحيحة أو خاطئة - هذا ليس اختبارً.**
2. **يرجى الإجابة على جميع الأسئلة بصدق ودقة بقدر ما تستطيع - وهذا مهم جدا.**

1. النشاط البدني في وقت فراغك: هل قمت بأي من الأنشطة التالية خلال الـ 7 أيام الماضية (الأسبوع الماضي)؟ إذا كان الجواب نعم، كم مرة؟ (ضع علامه √ فقط لكل صف.)

نوع النشاط	لاشئ	1-2	4-3	5-6	7 أو أكثر
الوثب بالحبل					
التزلج بالعجلات					
لعبة المسّاكة					
المشي للتمرين					
ركوب الدرجات					
الركض أو الجري					
التمرينات الهوائية أو العَرَابَة					
السباحة					
التنس					
الرقص					
كرة القدم					
تنس الريشة					
التزلج بالبورد					
الهوكي					
كرة السلة					
فنون الدفاع عن النفس والكاراتيه					
أخري :					

2. في الأيام السبعة الأخيرة ، أثناء فصول (حصة) التربية البدنية (الالعاب) ، كم عدد المرات التي كنت فيها نشيطًا للغاية (اللعب بقوة ، الجري ، القفز ، الرمي)؟ (أختار واحد فقط.)

أ) لا اخذ هذا الفصل
ب) بالكاد
ج) في بعض الأحيان
د) في كثير من الأحيان
ه) دائما

3. في الأيام السبعة الماضية ، ماذا فعلت معظم الوقت في العطلة؟ (إختار واحد فقط.)

أ) جلست (للتحدث وللقراءة وللقيام بالواجب المدرسي)
ب) قمت بالمشي
ج) لعبت وركضت قليلا جدا
د) لعبت وركضت بعض الوقت
ه) لعبت وركضت كثيرا

4. في الأيام السبعة الماضية ، ماذا كنت تفعل عادة في الغداء (بالإضافة إلى تناول الغداء)؟ (أختار من واحد فقط.)

أ) جلست (للتحدث وللقراءة وللقيام بالواجب المدرسي)
ب) قمت بالمشي
ج) لعبت وركضت قليلا جدا
د) لعبت وركضت بعض الوقت
ه) لعبت وركضت كثيرا

5. في الأيام السبعة الأخيرة ، كم عدد الأيام بعد المدرسة مباشرة قمت بممارسة الرياضة أو الرقص أو ممارسة الألعاب التي كنت فيها نشيطًا للغاية؟ (أختار واحد فقط.)

أ) ولا مرة
ب) مرة واحدة خلال الأسبوع السابق
ج) مرتين أو ثلاث مرات خلال الأسبوع السابق
د) أربع مرات خلال الأسبوع السابق
ه) خمس مرات خلال الأسبوع السابق

6. في الأيام السبعة الأخيرة ، كم عدد المرات قمت فيها بالرياضة فى المساء ، أو الرقص ، أو ممارسة الألعاب التي كنت فيها نشيطاً للغاية؟ (أختار واحد فقط.)

أ) ولا مرة
ب) مرة واحدة خلال الأسبوع السابق
ج) مرتين أو ثلاث مرات خلال الأسبوع السابق
د) أربع أو خمس مرات خلال الأسبوع السابق
ه) ستة أو سبعة مرات خلال الأسبوع السابق

7. في عطلة نهاية الأسبوع الماضي ، كم عدد المرات التي مارست فيها الرياضة ، أو الرقص ، أو ممارسة الألعاب التي كنت فيها نشيطًا للغاية؟ (أختار واحد فقط.)

أ) ولا مرة
ب) مرة واحدة
ج) مرتين أو ثلاث مرات
د) أربع أو خمس مرات
ه) ستة أو أكثر

8. أي مما يلي يصف لك أفضل شيئ خلال السبعة أيام الماضية؟ اقرأ جميع العبارات الخمس قبل أن تقرر الإجابة الوحيدة التي تصفك

أ) أقضى كل أو معظم وقت فراغي في عمل أشياء تنطوي على جهد بدني قليل.

ب) في بعض الأحيان من 1 - 2 مرات في الأسبوع الماضي فعلت أشياء تعتمد عل مجهود بدنى في وقت فراغي .

ج) في بعض الأحيان من 3 - 4 مرات في الأسبوع الماضي فعلت أشياء تعتمد عل مجهود بدنى في وقت فراغي .

د) في بعض الأحيان من 5 - 6 مرات في الأسبوع الماضي فعلت أشياء تعتمد عل مجهود بدنى في وقت فراغي .

د) في كثير من الأحيان من 7 مرات او أكثر في الأسبوع الماضي فعلت أشياء تعتمد عل مجهود بدنى في وقت فراغي .

9. أوصف عدد المرات التي مارست فيها النشاط البدني (مثل ممارسة الألعاب الرياضية أو الألعاب أو الرقص أو أي نشاط بدني آخر) لكل يوم في الأسبوع الماضي.

الأيام	أبدا	نادرا	متوسطه	غالبا	بإستمرار
الجمعة					
السبت					
الأحد					
الأثنين					
الثلاثاء					
الأربعاء					
الخميس					

10 . هل كنت مريض / مريضة في الأسبوع الماضي ، أو هل منعك أي شيء من القيام بأنشطتك البدنية الطبيعية؟ (ضع علامة على واحدة.)

نعم .... لا ....

فى حالة الإجابة بنعم ، أذكر السبب الذى منعك

....
